# X-ray micro-computed tomography in willow reveals tissue patterning of reaction wood and delay in programmed cell death

**DOI:** 10.1186/s12870-015-0438-0

**Published:** 2015-03-11

**Authors:** Nicholas James Beresford Brereton, Farah Ahmed, Daniel Sykes, Michael Jason Ray, Ian Shield, Angela Karp, Richard James Murphy

**Affiliations:** Institut de recherche en biologie végétale, Université de Montréal, Montreal, QC H1X 2B2 Canada; Micro-CT Lab, Imaging and Analysis Centre, Natural History Museum, London, SW7 5BD UK; Department of Chemistry, Imperial College, London, SW7 2AZ UK; Department of AgroEcology, Rothamsted Research, Harpenden, Herts AL5 2JQ UK; Centre for Environmental Strategy, University of Surrey, Guildford, Surrey GU2 7XH UK

**Keywords:** Willow, Biofuel, X-Ray micro-computational tomography, Programmed-cell-death, Reaction wood

## Abstract

**Background:**

Variation in the reaction wood (RW) response has been shown to be a principle component driving differences in lignocellulosic sugar yield from the bioenergy crop willow. The phenotypic cause(s) behind these differences in sugar yield, beyond their common elicitor, however, remain unclear. Here we use X-ray micro-computed tomography (μCT) to investigate RW-associated alterations in secondary xylem tissue patterning in three dimensions (3D).

**Results:**

Major architectural alterations were successfully quantified in 3D and attributed to RW induction. Whilst the frequency of vessels was reduced in tension wood tissue (TW), the total vessel volume was significantly increased. Interestingly, a delay in programmed-cell-death (PCD) associated with TW was also clearly observed and readily quantified by μCT.

**Conclusions:**

The surprising degree to which the volume of vessels was increased illustrates the substantial xylem tissue remodelling involved in reaction wood formation. The remodelling suggests an important physiological compromise between structural and hydraulic architecture necessary for extensive alteration of biomass and helps to demonstrate the power of improving our perspective of cell and tissue architecture. The precise observation of xylem tissue development and quantification of the extent of delay in PCD provides a valuable and exciting insight into this bioenergy crop trait.

**Electronic supplementary material:**

The online version of this article (doi:10.1186/s12870-015-0438-0) contains supplementary material, which is available to authorized users.

## Background

Dedicated bioenergy crops have the potential to provide a sustainable and carbon neutral replacement to petroleum based liquid transport fuels. However, the glucose rich cell walls of dedicated bioenergy crops (such as willow or *Miscanthus* in the UK) are generally recalcitrant to deconstruction, requiring high amounts of energy and severe chemical pretreatment before the glucose can be released in a form suitable for fermentation. To overcome this barrier, research efforts worldwide have been directed towards understanding the natural variation of cell wall recalcitrance in dedicated bioenergy crops.

The basis of genotype-specific variation in recalcitrance was recently identified in the fast-growing biomass crop willow (*Salix sp.*) as genetic variation in a natural response to *gravity*, known as the “reaction wood” (RW) response [1]. RW formation in trees is characterised by major alterations in xylem cell development and tissue patterning in the stem in response to displacement from vertical, either through the perception of gravity or mechanical load. These changes are polarized across the stem with the “upper” side of the stem termed Tension Wood (TW) and the “bottom” side termed Opposite Wood (OW). Despite being recognised as a key determinant of glucose yield, many aspects of this trait, and specifically how the trait differs between genotypes to result in such large alterations to glucose release yields, remains a mystery.

### General reaction wood tissue patterning and development

The majority of tree biomass develops from the vascular cambium, the ring of differentiating cells between the bark and the inner/secondary xylem. The proportion of the secondary xylem to the biomass of the stem varies with age and genotype, but is roughly 85-90% [2]. Most angiosperms, such as willow (*Salix* sp.), have a degree of specialisation within the secondary xylem, with fibre cells predominantly delivering the structural demands of the organism, vessel elements comprising purely hydraulic architecture and ray parenchyma cells thought to mostly serve as storage elements. This increased tissue complexity and diversity of function is distinct from the more ancient gymnosperms, where tracheids serve both functions.

Further specialisation has evolved in a smaller number (<50%) of woody angiosperms [3] where gelatinous fibres (g-fibres) can form on the TW side of secondary xylem, in a stem displaced from vertical, in order to return the apical meristem to vertical and increase the mechanical strength of the stem. The structural re-enforcement of fibre cells with an extra cell wall layer (the gelatinous layer or g-layer) is developed at the expense of the fibre cell lumen, and thus chould be accompanied by a deleterious reduction in water conductance in TW. A positive correlation between fibre cell lumen and xylem water capacity has been observed by Pratt *et al.* [4]. Even though there is this large change in cell structure upon RW formation, most g-fibre forming angiosperms, unlike gymnosperms [5,6], are thought to maintain their efficient water translocation, although the mechanism of how this is achieved is unclear.

During secondary xylem development from the vascular cambium, normal fibre cells undergo a very strictly controlled apoptosis, the end result being long tube-like cells with thick secondary cell walls and no protoplast. How the process of programmed-cell-death (PCD) is altered in TW development is poorly established in terms of evidence, but it has been suggested in several reviews [7,8] that PCD is delayed in certain species of poplar, with this delay hypothesised as being necessary to accommodate g-layer biosynthesis.

### X-Ray micro-computed tomography (μCT)

X-Ray μCT has been used increasingly as a powerful method for plant anatomical assessment mainly driven by its value in the timber industry for evaluation of wood quality. Recent published studies, while low resolution in terms of the current state-of-the-art, show how this non-destructive technology can be used systematically to identify the presence or absence of rameal traces, i.e. irregularities relating to branching such as knots, in oak [9]. High resolution X-Ray μCT has, over the past decade, been presented as a potentially valuable method for quantitative investigation of plant anatomy in numerous studies, and more recently wood anatomy. Stuppy *et al.* [10] demonstrated how 3D architecture could be rendered (at a relatively poor linear resolution of 50 μm) in a diverse range of plants including sections of palm, oak, pineapple, a tulip flower and inflorescence of *Leucospermum tottum*. Exclusively in wood, broad tissue 3D models have been rendered of sections of: beech, oak, spruce heartwood, Douglas fir, loblolly pine, teak and eucalyptus (as well as non-woody Arabidopsis) [11-13]. Most recently Broderson *et al.* have established a range of tools useful for assessment of 3D xylem structre using X-Ray μCT [14,15].

### Variation in reaction wood

Juvenile willow genotypes (3 month old) grown under greenhouse conditions only exhibit fully mature field (3 year old trees, 7 year root stock) lignocellulosic sugar yield phenotype if tipped to induce RW [1], demonstrating the significance of variation in RW response to wood development as well as the constant RW inducing conditions of field environments. It seems likely that the high sugar release yields achieved from willow and poplar biomass is due to abundance of the cellulose rich g-layers in TW tissue of RW (which are always present to some extent in short rotation coppice (SRC) willow stem sections) and that sugar release yield variation between genotypes is therefore due to variation in g-fibre abundance. Evidence for this is absent to date and, surprisingly, some genotypes of willow that do not significantly increase in sugar release upon RW induction did have increased g-fibre abundance [1]. This suggests that variation in RW might extend beyond g-fibre abundance alone. Traditional sectioning and microscopy fall short of providing a means of robust quantification of RW tissue patterning on a whole tree level as a transverse section or several transverse sections may not be representative due to the irregular nature of wood growth. To overcome these limitations, an approach to larger scale 3D tissue assessment was devised in the hope further resolving the nature of the RW response.

To test the hypothesis that tissue patterning alters significantly upon RW induction; we used 3D X-Ray μCT to directly assess wood architecture in willow trees after being grown vertically and *tipped* at 45°.

## Methods

### Plant cultivation and RW induction

Six short rotation coppice willow cuttings (cultivar Resolution – pedigree: (*S. viminalis*. x (*S. viminalis*. x *S. schwerinii* SW930812)) x (*S. viminalis*. x (*S. viminalis*. x *S. schwerinii* ‘Quest’))) were planted in 12 l pots with 10 l of growing medium consisting of ^1^/_3_ vermiculite, ^1^/_3_ sharp sand and ^1^/_3_ John Innes No.2 compost, by volume. Trees were then grown under a 16 h (23°C) day cycle and an 8 h (18°C) night cycle for 12 weeks. After 6 weeks of growth all stems from all trees were tied to a supporting bamboo cane at regular intervals and three of the trees tipped at a 45° angle to the horizontal (three left growing vertically as controls). All trees were checked every two days and tied to maintain controlled growth orientation, either 45° or vertical. After 12 weeks of growth (and 6 weeks of differential treatment) tree stem biomass was harvested.

### Fixation, sectioning, staining and microscopy

Upon stem harvest an eight cm section of the measured middle of all the stems from each tree was debarked (for ease of 2° xylem specific analysis) and “fixed” in FAA (formaldehyde 3.7%, acetic acid 5% – ethanol 47.5%). The fixation step is crucial to maintain cell contents for downstream 3D X-ray μCT. Sections were then cut into four cm sections, one was air dried for 3–5 days before X-ray μCT, whilst the other was used for sectioning (using a sledge microtome to 25 μm) and histochemisty before then being used for destructive basic density assessment. Sections were stained with 1% safranin O (aq) as an unspecific cell wall counterstain, 1% chlorazol black E (in methoxyethanol) to stain g-layers [1,16] or with 1% Coomassie to highlight the remnants of cell content. As fibre cell length can often be greater than 1 mm (and sections for microscopy were limited to 25 μm depth) efforts were made to compare these partial cell images to 3D data. Further comparative analysis was conducted using cell wall and cell contents auto-fluorescence confocal microscopy by Z-stacking with high resolution for closer comparison of cell content fragments to 3D images. Excitation and emission wavelengths were 488 nm and 500-700 nm respectively [17].

### Basic density assessment

The basic density of wood was assessed using traditional methods [18]. Here, 2-4 cm wood sections were vacuum infiltrated with water before green volume was measured via water displacement and wood oven dry weight was measured after drying over night at 105°C.

### X-ray μCT scanning

The scans were performed using a Nikon Metrology HMX ST 225. The samples were scanned using a tungsten reflection target, at an accelerating voltage of 160 kV and current of 180 μA using a 500 ms exposure time (giving a scan time of 25 minutes). No filters were used and 3,142 projections were taken over a 360° rotation. The voxel size of the resulting dataset had linear resolution of 9 μm. A common piece of willow wood, cut from NW of control trees and treat in the same manner as the samples, was used as a reference standard and scanned alongside each sample. For 2D images, high voxel intensity, and therefore greater X-Ray attenuation, is visible as lighter regions whereas regions of low voxel intensity are visible as darker regions.

### 3D image processing

The 3D volumes were reconstructed using CT Pro (Nikon Metrology, Tring, UK) and TIFF stacks exported using VG Studio Max (Volume Graphics GmbH, Heidelberg, Germany) (see Additional file 1). Drishti [19] was used to generate a 3D rendering and analysis of ROI. A standardised transfer function was designed and applied to each 3D ROI in isolation to allow comparison. ROI were also saved as individual 2D Tiff files and MatLab (MATLAB 6.1, The MathWorks Inc., Natick, MA, R2012b) was used to collate data from all files and produce histograms of voxel intensity distribution. The reference standard was used to normalise voxel intensity and allow direct comparison of collated data between samples.

## Results

### Density and G-layer verification

**Figure 1:**
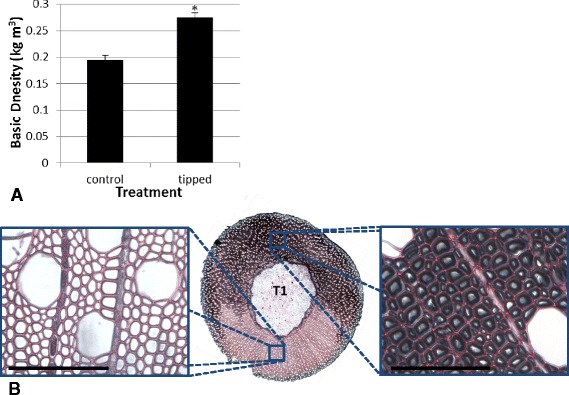
**Reaction wood impact on basic density and 2D xylem architecture.** **A** Basic density of debarked willow cultivar Resolution after 3 months of growth either unperturbed or including 6 weeks of RW induction (tipping at 45° from vertical). n = 3 trees. **B** Transverse middle stem section (25 μm) of a RW induced tree stained with safranin O (red – nonspecific staining the cell wall) and chlorazol black (black – specifically staining the g-layer of g-fibres). Panels: OW (left) and TW (right) are included with scale bar = 100 μm. *p < 0.05 (Students t-test).

The basic density of 2–4 cm long stem middle segments from 3 month old willow (cultivar Resolution) was assessed after trees had been tipped for 6 weeks of their growth or grown vertically as controls. Debarked stem segments from control trees had an average basic density of 195 kg/m^3^ which was significantly (t-test p < 0.001) increased in similar segments from RW induced trees, to 275 kg/m^3^ (Figure 1A). Stems were then sectioned and stained which confirmed that RW induction had successfully produced g-fibres (Figures 1B). RW induced trees had abundant g-fibre production with clear transverse polarisation aligned with the vector of gravitational stimulus (“upper” stem during tipping).

### μCT scanning and voxel intensity/distribution of regions of interest

**Figure 2:**
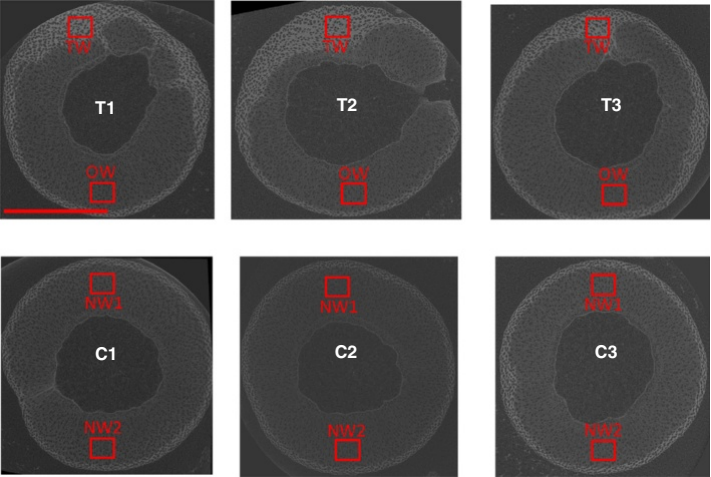
**2D transverse X-Ray CT scans.** A single representative image from the stack reconstructed from the X-ray CT scanning of each stem segment. Each tree is either RW induced (T1, T2 and T3) or a control grown without induction (C1, C2 and C3). Regions of interest assessed for voxel intensity and distribution, TW, OW and NW are highlighted in red. High voxel intensity, and therefore 554 great X-Ray attenuation, is visible as lighter regions whereas regions of low voxel intensity are visible as darker regions. Scale bar = 4mm.

Reconstruction of μCT scans from each of the six stems were made allowing generation of ~1500 (2 MB) images each. These images were then stacked and rendered into a 3D volume where the stem segment is reproduced *in silico* down to a voxel (a 3D pixel) representing an *in planta* linear resolution of 9 μm. Clear increases in X-ray attenuation (represented by voxel intensity) were visible at the lateral part of the stem, corresponding anatomically to the vascular cambium, elongating secondary xylem and maturing secondary xylem tissue (Figure 2). In TW, this region of increased voxel intensity was greatly extended, also from the periphery of the stem, and with transverse polarisation aligned with the vector of gravitational stimulus (Figure 2*top three panels*).

**Figure 3:**
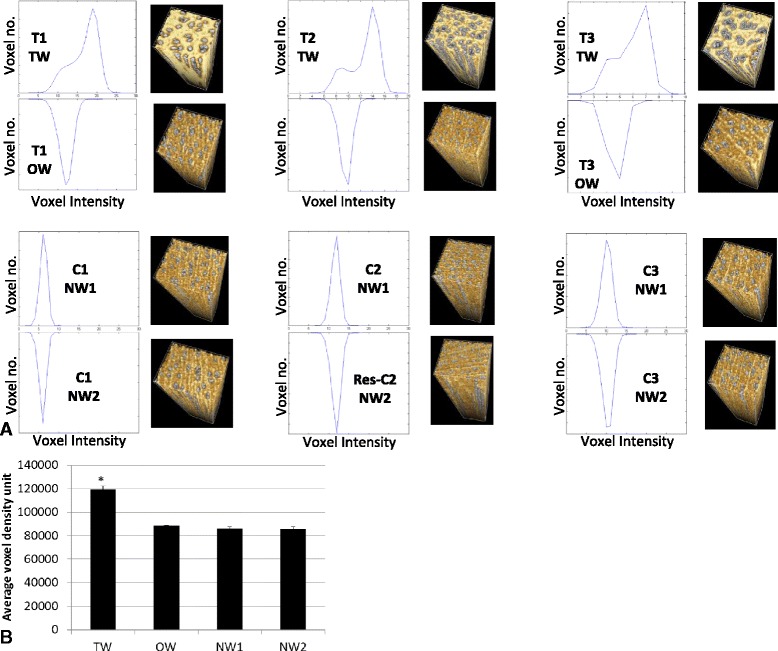
**Voxel distribution.** **A** Matlab histograms of voxel intensity distribution for each ROI, TW, OW and NW and 3D render of each ROI, units are not included as the number of voxels varied (histograms are to compare intensity distribution). Each tree, RW induced (T1, T2 and T3) or controls (C1, C2 and C3) were scanned including a common internal standard – allowing comparison of average voxel intensity. **B** Average ROI voxel intensity. Error bars = standard error of tissue type across 3 trees. * *p* < 0.05 (one-way ANOVA).

The 3D Regions Of Interest (ROI) were then isolated *in silico* representing: TW, OW and NW (Figure 2). These ROI were assessed for voxel intensity and spatial distribution. Using MatLab, histograms of the voxel intensity for each ROI were plotted (n ranging from 3–14 million voxels for each ROI) (Figure 3). The distribution of voxels was consistent between OW and NW but distinct for TW. The voxels for a given ROI were then each counted into one of 26 bins of relative greyscale intensity from 0–50000 (0–1999, 2000-3999…) with numbers of voxels in each bin expressed as a proportion of total ROI voxels. The relative bin greyscale intensity was then normalised against a small segment of willow used as a common internal standard for each scan. When the normalised average voxel intensity of each scan was compared, TW was the only tissue to be significantly increased (Figure 3B, t-test p < 0.05).

### Treatment specific tissue patterning/architectural patterning

**Figure 4:**
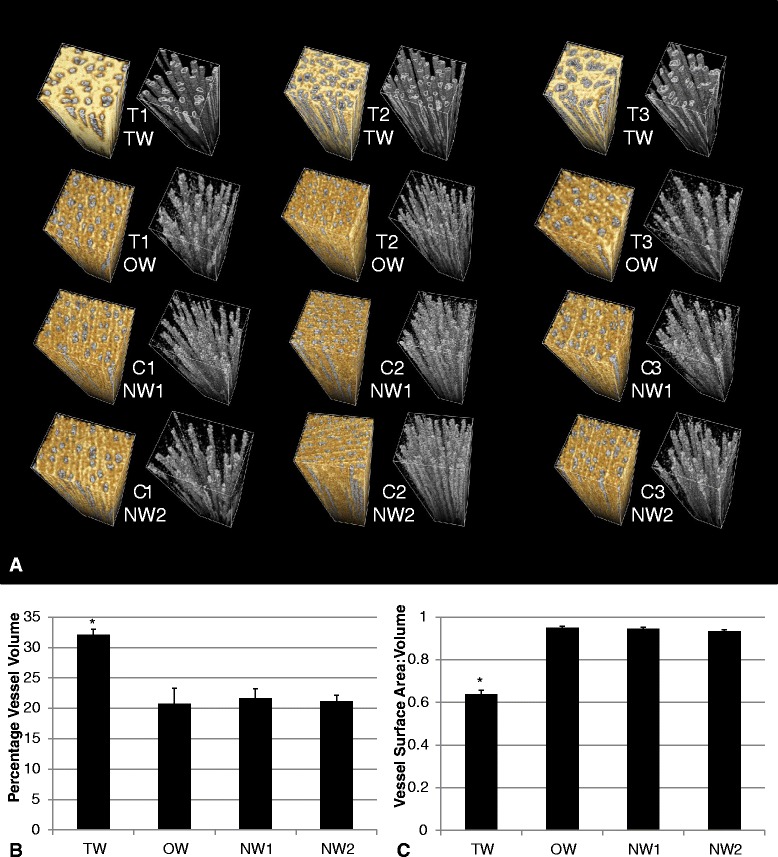
**3D xylem architecture. A** 3D render of each ROI (TW, OW or NW) from X-ray CT scans of RW induced trees (tipped T1, T2 and T3) or controls (C1, C2 and C3). The 3D ROI render on the right after the common vessel specific transfer function was applied *in silico*. **B** Total volume of vessels as a percentage of each ROI was averaged for each tissue. **C** Vessel surface area:volume ratio of each ROI was averaged for each tissue. Error bars = standard error of tissue type across 3 trees. * *p* < 0.05 (one-way ANOVA).

As well as quantifying average voxel intensity, voxels can be binned according to intensity *in silico,* this can be applied to each rendered volume using a common transfer function as part of the image processing [19] to quantify (and view) voxel groups of similar intensity. In this way it was possible to isolate the vessel elements within each tissue type to compare architectural changes generated by RW induction (Figure 4).

The vessel frequency was consistent between the OW and NW ROI (averaging 37 vessel elements) but reduced in the TW ROI (averaging 30 vessel elements). However, vessel *volume* was significantly increased by over 50% in the TW ROI (Figures 3 and 4). The total vessel surface area (per cm^3^) can also be quantified after isolation using the common transfer function; in tension wood the vessel surface area to vessel volume ratio drops well below an average of 0.9-0.95 to that of 0.64 (the largest cell type present in the stem).

### Quantification of delayed programmed cell death

**Figure 5:**
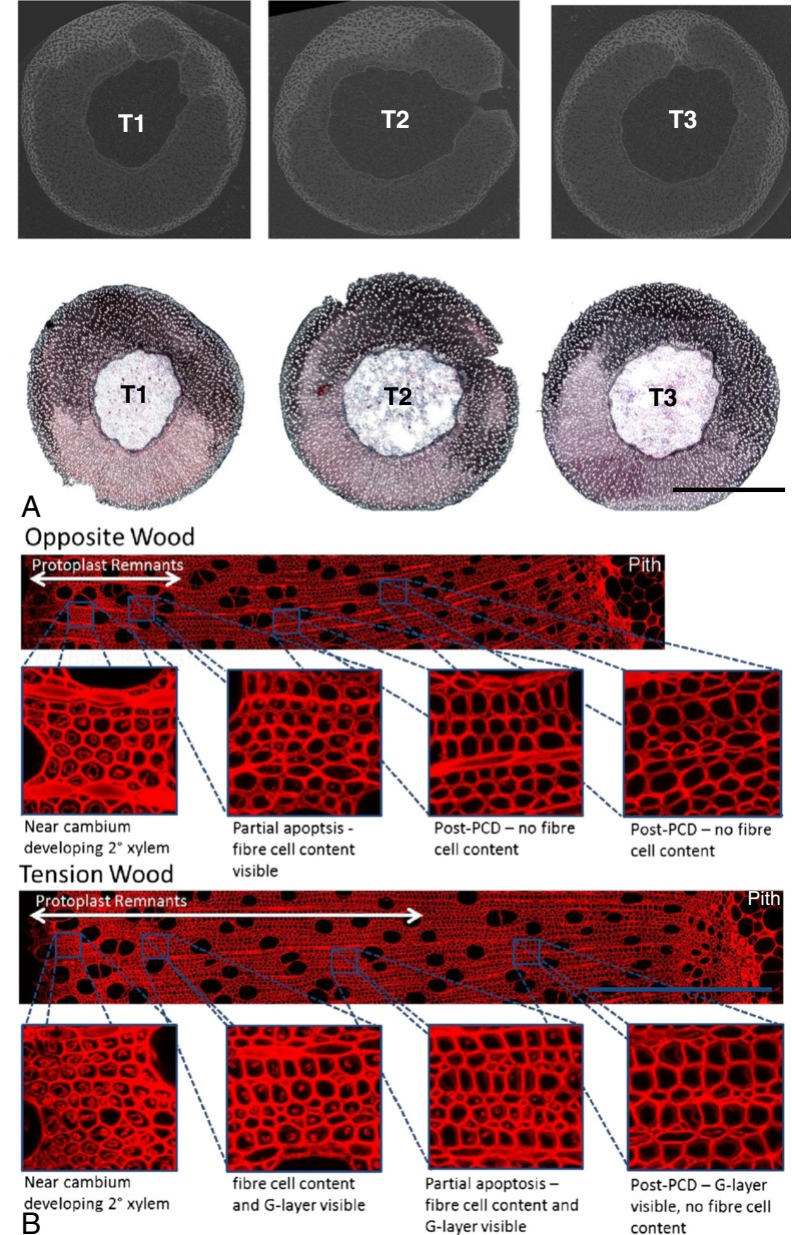
**Tension wood delay in programmed-cell-death. A** Top, single representative images from the stack reconstructed from the X-ray CT scanning of each RW induced stem segment. Bottom, Transverse middle stem section (25 μm) of a RW induced tree stained with safranin O (red – non-specific staining the cell wall) and chlorazol black (black – specifically staining the g-layer of g-fibres). Scale bar = 4 mm. **B** Confocal micrograph of coomassie stained OW (top) and TW (bottom), autofluorescence is shown in red (excitation and emission wavelengths were 488nm and 500-700nm respectively). Panes highlight the difference between OW fibre and TW g-fibre development in terms of individual cell structure and greater tissue architecture in relation to the whole stem. Blue scale bar = 500 μm.

The variation in X-ray attenuation, and so voxel intensity, observed in RW induced trees was clearly aligned with TW but did *not* correspond to g-fibre presence in the tissue (Figure 5). This is not surprising on a cell by cell basis as resolution was not great enough to distinguish individual fibre cells (but was sufficient to distinguish vessel elements) despite the fact that each voxel was resolved to 9 μm.

Interestingly, the pattern of increased voxel intensity followed that of developing xylem before the termination of fibre cell maturation and completion of PCD. The post-cambial cells, from the secondary xylem elongation stage to the onset of autophagy where the protoplast and cytoplasmic contents are still retained, was visible as a circle surrounding the stem present in both control and RW induced trees.

The delay of PCD often referred to as associated with TW can be seen by light microscopy, but is inadequately represented due to the transverse sectioning process. By using direct coomassie staining and Z-stacked confocal microscopy of 25 μm sections the extension of cell life can be roughly observed in TW (Figure 5B). Fibre cells are sheared during the sectioning process leaving only a proportion of the cell contents/remnants visible so that, whilst the irregular nature of this extended tissue patterning is evident, quantification is difficult. By assessing this tissue patterning without sectioning, by X-ray μCT, the extent of this irregularity was revealed directly (Figures 2 and 5).

## Discussion

RW response has been identified as a principle cause of variation in enzymatic saccharification yields in willow, yet understanding of the tissue architectural and cellular remodelling associated with RW has typically been limited to classical sectioning and microscopy. Here, RW stem remodelling was explored using μCT following the theory that such widespread alterations, with accompanying extensive influence on saccharification yields, would likely effect enough change in X-ray attenuation as to be amenable to more direct quantification.

### Density, G-layer verification and distribution of voxel intensity

The tipping of trees at 45° and maintaining this angle by restraint is designed as an *analytical* technique for studying RW. RW induction is not optimised to produce large amounts of TW but to deliver a stimulus in a consistent and controlled manner allowing transferable analysis of the response. This constant, known magnitude of stimulus is crucial to such studies as, in field-grown willow trees, g-fibres can always be seen in transverse sections willow material. The explanation for this is that trees in the field are constantly exposed to some degree of RW inducing stimulus from the environment but of an ever-changing intensity and from varying vectors in the form a wind speed, land incline and/or internal growth stresses [1]. A number of common morphological alterations have been reported to occur in both Poplar and willow upon development under increased RW inducing conditions, either gravitational or thigmomorphogenic in nature. A common result is more compact growth, with reduced stem height, increased diameter and increased density [20,21]. These make sense from an architectural standpoint as, under conditions such as high wind speeds, the structure of a smaller, wider stem will reduce the stress a stem is exposed to. The degree to which such changes vary between different varieties has been less well studied.

The utilisation of the RW response is an attractive way to increase sugar accessibility in willow due to being part of natural plant physiology, and so unlikely to negatively impact plant integrity. In fact, large increases in sugar yield have been reported without any detriment to biomass yield (although only in pot trials) even though plant size was reduced [1,22]. An increase in density straightforwardly describes this phenomenon and speaks to the extent of the changes in biomass structure elicited during RW formation. The density observed in the pot grown trees here (195 and 275 kg/m^3^) is very low when compared to that of mature willow (~300 – 500 kg/m^3^) [23], but not surprising for juvenile, debarked wood. The increase in density associated with RW induction may be due to increased g-fibres, which substantially increase in abundance upon induction (Figure 1), as the extra cell wall layer replaced fiber lumen void space. Whilst g-fibre abundance was clearly increased, g-fibre enriched TW was not visible in the μCT scans, this is likely due to the linear resolution of the scans which was just short of a fibre cell width (once voxel bleeding, a localised overlap of signal, is accounted for) at ~10 μm as well as due to the nature of the extra cell wall layer (g-layer), which did not greatly attenuate X-rays being almost entirely composed of cellulose. However, clear differences in X-ray attenuation associated with RW induction were observable in 3D.

### Treatment specific tissue patterning/architectural patterning

Broad secondary xylem tissue remodelling occurs during RW formation. An increase in vessel length but severe decrease in vessel frequency in tension wood of young inclined stems was recorded in poplar [24] and consequently total vessel volume should be reduced as a product. Our data agrees with this reduction in vessel frequency but also measures the volume of vessels in relation to other cell types in the xylem. As can be seen in Figure 4, the total volume of vessels is greatly increased in tension wood of the willow variety Resolution, even though the frequency is reduced. From this we can speculate that there is no penalty to trees grown in high RW inducing conditions due to limitations in water transport capacity. This increase in vessel volume:surface area ratio, in certain parts of tension wood, may represent a mechanism by which this maintenance of conductivity is achieved and may also reflect the penalty associated with such structural change if an increase in volume is required due to a reduction in efficiency of the new vessel structure.

The increase in relative fibre cell frequency is structurally necessary to either bring the stem back to vertical or help tolerate the increased load bestowed by the displaced stem. This remodelling is made at the expense of vessel number, yet a reduction in the water transporting capacity would be detrimental to plant fitness. It is not then surprising to see alterations to vessel dimensions to mitigate this penalty. Jourez *et al.* [24] also found that solitary vessels in TW, whilst less circular, had a greater external diameter (2 μm more) and greater length (10 μm more). When these increases are envisaged in 3D, the volume of vessels is likely to be larger. This would agree with another of their findings that mean lumen of TW vessels is larger (5%) than OW. Remodelling resulting in such large increases in vessel volume suggests that the TW form may not be as efficient at water transport but is still effective as well as permitting a greatly increased structural function. They also discuss the variability of such measurements in different species and we would reiterate that these changes are likely to be species and variety specific.

This trade-off between mechanical support and water conductivity is recognised in conifers as compression wood has reduced *k*_*s*_ (specific water conductivity) [5,6]. Unlike angiosperms, gymnosperms, a more ancient phylum in evolutionary terms, do not have specialised vessel cells so the homogenous tracheids play the role of bestowing large structural modification without loss of plant integrity alone. Gartner *et al.* [25] found that *Quercus ilex* (holm Oak) TW elicits large scale modification for mechanical support *without* impairment to water conductivity (specific conductivity, *k*_*s*_). Interestingly for Gartner *et al.* [25], whilst vessel area was similar, vessel frequency was actually increased in tension wood – the reverse physiological solution to that implied by the tissue modifications here in willow but with the same outcome. The lesson from nature here is that complex interdependent relationships exist between biomass mechanics support and water conductivity, or importantly from a bioenergy perspective, between lignocellulosic sugar yield (as driven by RW) and water-use-efficiency (of great importance for crop sustainability).

A reduction in vessel lumen area in poplar tension wood has been well documented [20,21,26] and is in contradiction to the data revealed here for willow. Whilst this may be a point of distinction between willow and poplar, the ROI specifically investigated, or novel method of their assessment here, may also be the source of this disparity. The ROI were selected specifically as the regions of variation in terms of X-ray attenuation which were located at the periphery/lateral side of the stem (Figure 2). We can see that at this periphery tissue architecture is distinct from more medial/older wood of lower intensity, in a manner which is aligned with g-fibre orientation (the “upper” part of the stem) but not overlapping with g-layers. This variation within TW and lack of g-fibre associated increase in intensity was surprising. A major aim of the technique was to be able to separate fibres from g-fibres, therefore providing a valuable approach in quantifying g-fibre abundance in 3D (hopefully affording more accuracy than multiple transverse sections). Although resolution of the X-ray μCT fell short of such separation there was sufficient variation, associated with our treatment, to suggest TW tissue variation beyond g-layer presence alone. This led us to question what other TW tissue modifications might underlie such stark treatment specific differences as well as which might impact vessel size during development.

### Quantification of delayed programmed cell death

Although published evidence appears absent to date, it is well recognised that PCD is delayed in willow and poplar tension wood as fibre cell protoplast/cellular remnants can be observed (by microscopy) as present long after the completion of fibre cell maturation, apoptosis and degradation of cytoplasmic remnants in normal or opposite wood [7,8]. Overlapping RW formation and PCD EST libraries also strongly indicate alteration of “normal PCD” in xylem development of tension wood [27,28]. It is widely speculated that perhaps this delay occurs to accommodate biosynthesis of the g-layer from the plasma membrane, the extra internal cell wall layer of g-fibres, as the cellulose microfibrils would have to be produced after the establishment of the secondary cell wall.

Traditional methods of assessing cell *viability* are difficult to perform quantitatively in woody tissue as the process of transverse sectioning is such a destructive process, impairing methods such as NBT staining (for superoxide) and Tunnel Staining (for DNA degradation as resulting from autolysis). Whilst these methods are powerful in smaller model systems or when targeting limited numbers of cells, the assessment of larger scale tissue patterning in crops, such as the polarised patterning in wood as a result of RW induction, requires improved techniques for assessment and confidence.

Assessment is further made difficult by the natural asymmetry in wood growth (lack of perfectly uniform growth/cell development) as can be seen in Figures 1, 2 and 5. Hence, when investigating developmental variation due to RW formation a quantitative method, encompassing the variation across a given point in the stem but also the variation along the stem, is of substantial value. X-ray μCT provided a surprisingly powerful method for such assessment of PCD in 3D. Unlike histochemical or immunohistochemical 2D assessment of transverse sections where some amount of cell content is lost through processing, 3D analysis (if cell content is fixed/preserved as here) reveals the stark difference between fibre cells having completed PCD and those still developing or undergoing PCD. This is, in retrospect, predictable as the comparison in 3D is between “empty” fibre cells of secondary xylem and “solid” fibre cells retaining cell content/protoplast should reveal substantial difference in density. It is interesting to note that lines of increased voxel intensity, and so increased X-ray attenuation, are also visible leading from the pith of the wood to the periphery. Although these are not well resolved, they could potentially represent the ray parenchyma, which are also cells where cell content would be present and preserved.

Validation of the state of cell development in 2D (compared to single images of x-ray scans used for 3D rendering) was made using coomassie staining (data not shown) and z-stacked confocal microscopy (Figure 5B) of section made by traditional sectioning. It should be made clear that the nature of this assessment is by no means an assessment of *viability*, as methodology such as NBT staining but more an assessment of architectural variation which effects x-ray attenuation and, as such, is not limited to quantification of PCD.

One of the lessons evident from recent advances in biomass composition and enzymatic saccharification assessment is that above ground stem biomass varies between stems and throughout a stem and consequently should be considering in 3D. RW formation is a compelling illustration of this as it forms in localised positions across and along the stem at different times throughout biomass development, so that, if the genetics of a crop variety or the *net* composition of a feedstock for a biofuel process is to be elucidated, a holistic model of assessment is not only of great benefit but a necessity.

## Conclusions

Alterations to tissue patterning due to RW induction were visible in 3D using X-ray μCT. These changes describe the compromise or trade-off between hydraulic architecture and mechanical support. As the effectiveness of the functional interplay is likely to vary in a genotype specific manner, this RW remodelling would interestingly link dedicated bioenergy crop sustainability and yield via WUE and cell wall sugar accessibility. Greater resolution is needed to distinguish g-fibres and, as a more general property of the technique, the higher the resolution the more biological complexity can be investigated as there seems to be no “lower limit” to the patterning in nature. If resolution can be very slightly increased while the macro scale of biological samples is maintained then X-ray μCT could join contemporary *in vivo* imaging techniques such as GFP-fusions.

Variation in RW response has been established and identified as being of major importance to glucose yields from willow biomass, however, the root cause of this variation remains unclear. Evidence of variation in g-layer structure has been shown to exist between species [29]. Further work should focus on quantitatively assessing the degree to which, if any, delay in PCD varies between genotypes known to differ in RW response in terms of glucose yield. If the basis for the delay in PCD is in fact due to a necessity of maintaining the protoplast for g-layer construction, variation in the extent of this delay may affect the composition, structure and/or abundance of the layer, and consequently be of key importance to lignocellulosic biofuel yields.

### Availability of supporting data

The video supporting the results of this article is available in the BMC-series YouTube channel repository, http://youtu.be/CxWR10gdwQc.

## Additional file

Additional file 1:
**3D video rendered from X-Ray μCT scans of willow, cultivar Resolution.** Stem segment (2 cm length) was air dried for several months without fixation, bark was retained. Void volume of the wood was coloured in blue to highlighted vessel element lumens.
